# Systematic druggable genome-wide analysis to identify potential therapeutic targets for urologic diseases

**DOI:** 10.1016/j.gendis.2025.101922

**Published:** 2025-11-08

**Authors:** Yongwang Shi, Hongyan Chen, Xinlei Zhang, Chengliang Yin, Penghu Lian

**Affiliations:** Department of Pediatrics, Peking Union Medical College Hospital, Chinese Academy of Medical Sciences & Peking Union Medical College, Beijing 100730, China; 4+4 Medical Doctor Program, Chinese Academy of Medical Sciences & Peking Union Medical College, Beijing 100730, China; Beijing ClouDNA Technology Co., Ltd., Beijing 100029, China; Medical Innovation Research Department, Chinese PLA General Hospital, Beijing 100853, China; Department of Urology, Peking Union Medical College Hospital, Chinese Academy of Medical Sciences & Peking Union Medical College, Beijing 100730, China

Urologic diseases, including benign and malignant conditions, impose a major global health burden.^1^ However, therapeutic development remains limited by an incomplete understanding of causal molecular drivers.^2^ Here, we performed a genome-wide Mendelian randomization analysis integrating blood-derived expression quantitative trait loci (eQTL) with genome-wide association study (GWAS) data, focusing on druggable genes with cis-eQTL evidence to systematically prioritize and validate therapeutic targets for urologic diseases (Fig. S1). Through two-sample Mendelian randomization, we identified genes with putative causal roles in urologic diseases, including leukocyte receptor tyrosine kinase (LTK) for bladder cancer and cyclin A2 (CCNA2) for benign prostatic hyperplasia (BPH). Functional annotation revealed key pathways and protein–protein interaction networks relevant to disease pathogenesis. Both genes demonstrated potential clinical relevance in external datasets. These findings provide genetically supported targets with translational potential and may contribute to the development of more effective treatment strategies for urologic diseases.

To investigate causal links between blood-derived druggable gene expression and urologic diseases, we performed Mendelian randomization analyses using eQTLs from the eQTLGen consortium as instrumental variables and GWAS summary statistics for 24 urologic conditions from the UK Biobank (Table S1). After stringent instrument selection (*n* = 48,502 single nucleotide polymorphisms; median = 12 per gene), we applied the inverse-variance weighted (IVW) method and identified two genes that met the predefined criteria, while the remaining associations did not satisfy these thresholds. Higher genetically predicted expression of LTK was associated with a lower risk of malignant bladder neoplasms (odds ratio = 0.988; 95% confidence interval: 0.982–0.993; *P* = 5.15 × 10^−3^; Fig. 1A), while increased expression of CCNA2 correlated with elevated risk of prostate hyperplasia (odds ratio = 1.011; 95% confidence interval: 1.006–1.016; *P* = 4.22 × 10^−3^; Fig. 1A). Both genes were supported by strong instruments (F-statistics > 10) and showed no evidence of heterogeneity (*P* = 0.424 for LTK; *P* = 0.570 for CCNA2).

**Figure 1:**
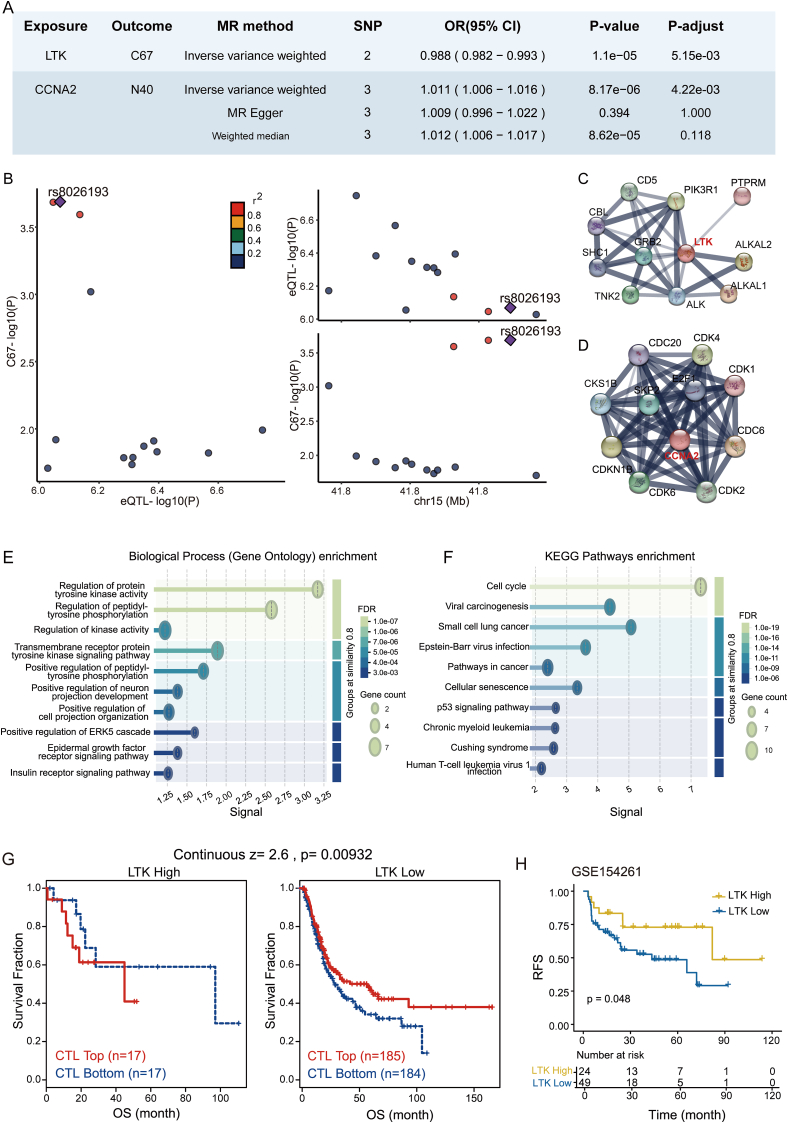
Mendelian randomization analysis between blood-derived expression quantitative trait loci (eQTLs) and urologic diseases, and validation of druggable genes. **(A)** Mendelian randomization analysis revealed a significant association between *LTK* and bladder cancer, as well as between *CCNA2* and benign prostatic hyperplasia. **(B)** Plot of the colocalization analysis between the eQTL of the *LTK* gene and malignant neoplasm of the bladder. *P*-adjust, *P*-value adjusted by Benjamini-Hochberg method; C68, malignant neoplasm of bladder; N40, benign prostate hyperplasia. **(C, D)** Protein–protein interaction (PPI) network of *LTK* (C) and *CCNA2* (D). **(E)** Enriched biological processes based on Gene Ontology for proteins in the PPI network of *LTK*. **(F)** Enriched KEGG pathways based on Gene Ontology for proteins in the PPI network of *CCNA2*. **(G)** Kaplan–Meier curves between high and low *LTK* expression groups, stratified into CTL Top and CTL Bottom in TCGA-BLCA. CTL, cytotoxic T lymphocyte. **(H)** Kaplan–Meier curves illustrate recurrence-free survival (RFS) in the GSE154261 dataset, stratified by high and low *LTK* expression groups.

To assess whether the observed Mendelian randomization associations were attributable to shared causal variants rather than linkage disequilibrium, we performed Bayesian colocalization analyses using single-nucleotide polymorphisms within ±100 kb of the transcription start sites. For LTK and bladder cancer, the posterior probability of a shared causal variant (PPH4) was 0.735, suggesting a likely common genetic basis (Fig. 1B). Similarly, CCNA2 and BPH demonstrated strong colocalization, with a PPH4 of 0.928, supporting a shared genetic signal underpinning the Mendelian randomization association (Fig. S2).

Database mining in DrugBank identified fostamatinib as a potential LTK inhibitor, although no known agonists were available. ClinicalTrials.gov records show that fostamatinib has been evaluated in ongoing or completed trials for multiple malignancies, including pancreatic ductal adenocarcinoma, renal cell carcinoma, non-small cell lung cancer, colorectal cancer, head and neck cancers, pheochromocytoma, and thyroid tumors.

To further investigate the biological relevance of the identified targets, we performed protein–protein interaction and functional enrichment analyses. For LTK, interacting partners, such as phosphoinositide-3-kinase regulatory subunit 1 (PIK3R1), anaplastic lymphoma kinase (ALK) and LTK ligand 2 (ALKAL2), and ALK, were identified (Fig. 1C), with Gene Ontology enrichment indicating roles in the regulation of protein tyrosine kinase activity, peptidyl-tyrosine phosphorylation, and receptor-mediated signaling (Fig. 1E), implicating LTK in oncogenic signaling relevant to bladder cancer. Similarly, CCNA2 was found to interact with key cell cycle regulators including cyclin-dependent kinase 1 (CDK1), S-phase kinase-associated protein 2 (SKP2), and cell division cycle 6 (CDC6) (Fig. 1D), and the enriched pathways were predominantly involved in cell cycle regulation and cellular senescence, both of which are mechanistically linked to prostatic hyperplasia (Fig. 1F).

We investigated the clinical significance of LTK using the Tumor Cancer Genome Atlas–Bladder Urothelial Carcinoma (TCGA-BLCA) and Gene Expression Omnibus (GEO) datasets. In the TCGA-BLCA cohort, LTK expression was found to be significantly positively correlated with cytotoxic T lymphocyte infiltration (*r* = 0.286; *P* = 4.99E-9; Fig. S3A), suggesting a potential link between LTK and immune infiltration. Based on the median expression level of LTK, patients were stratified into high and low expression groups (*P* < 2.2E−16; Fig. S3B). Further survival analysis revealed that in the LTK-low subgroup, patients with higher cytotoxic T lymphocyte infiltration exhibited significantly improved overall survival compared with those with lower cytotoxic T lymphocyte infiltration (*P* = 9.32E-3; Fig. 1G). This finding suggests that cytotoxic T lymphocyte infiltration may play a protective role in patients with lower LTK expression. To assess the clinical relevance of LTK in an independent cohort, we conducted Kaplan–Meier survival analysis using a GEO dataset GSE154261. The results demonstrated that patients with higher LTK expression had significantly better recurrence-free survival compared with those with lower expression (*P* = 0.048; Fig. 1H). This finding supports the potential prognostic value of LTK in bladder cancer and highlights its association with immune infiltration and disease progression. These results collectively indicate that LTK may serve as a key biomarker in bladder cancer, influencing both immune infiltration patterns and patient prognosis. Although data on BPH are limited, expression analyses of CCNA2 revealed consistent up-regulation in BPH compared with normal tissue, despite lacking statistical significance, suggesting a potential, though preliminary, role in disease development (Fig. S3C).

Bladder cancer ranks as the second most prevalent urologic cancer globally,^3^ yet its prognosis remains poor in advanced stages despite advances in chemotherapy and immunotherapy, underscoring the need to discover new therapeutic targets. LTK is a receptor tyrosine kinase, primarily expressed in neuronal cells and certain immune cells, including T-cells.^4^ Mendelian randomization analysis indicates that higher expression of LTK is causally associated with reduced bladder cancer risk, potentially through modulating cytotoxic T-cell infiltration, suggesting its value as an immune-related therapeutic target. BPH, a non-malignant enlargement of the prostate, is one of the most common urologic disorders in aging men and significantly affects quality of life^5^. However, its molecular mechanisms remain incompletely understood, limiting the development of targeted therapies. In our analysis, CCNA2, a gene regulating cell cycle progression, was identified as a putative causal gene for BPH via transcriptome-wide Mendelian randomization. Elevated expression of CCNA2 was associated with increased BPH risk, consistent with its known role in promoting cellular proliferation. These findings suggest that CCNA2 may serve as a potential therapeutic target for BPH, warranting further investigation into its function in prostatic tissue remodeling and hyperplasia. Given that the GWAS datasets used were predominantly based on European populations, the findings should be further validated in cohorts of other ancestries.

In summary, this study used two-sample Mendelian randomization to identify potential therapeutic targets for urologic diseases. The results revealed that genetically predicted expression of LTK was significantly associated with a reduced risk of bladder cancer, while CCNA2 expression was positively associated with an increased risk of BPH. By integrating Mendelian randomization analysis with colocalization, protein–protein interaction network analysis, and survival analysis, we demonstrated the potential of LTK and CCNA2 as clinically relevant therapeutic targets. Additionally, the association between LTK expression and immune infiltration highlights the role of immune modulation in bladder cancer pathogenesis. Future studies on LTK and CCNA2 are required to further elucidate their mechanistic roles in urologic diseases.

## CRediT authorship contribution statement

**Yongwang Shi:** Writing – original draft, Visualization, Validation, Formal analysis, Data curation. **Hongyan Chen:** Writing – original draft, Validation, Methodology, Investigation, Formal analysis, Data curation. **Xinlei Zhang:** Writing – review & editing, Supervision, Project administration. **Chengliang Yin:** Writing – review & editing, Supervision, Project administration. **Penghu Lian:** Writing – review & editing, Supervision, Project administration, Funding acquisition.

## Data availability

All data used in this study are publicly available. GWAS summary statistics (UK Biobank, round 2) were obtained from the Neale Lab (http://www.nealelab.is/uk-biobank/), eQTL data from the eQTLGen Consortium (https://eqtlgen.org/), and RNA-sequencing data for bladder urothelial carcinoma from TCGA (https://portal.gdc.cancer.gov/). Additional RNA-sequencing datasets were retrieved from GEO (GSE154261, GSE119195).

## Funding

This work was supported by the Chinese Academy of Medical Sciences Innovation Fund for Medical Sciences (No. 2023-I2M-C&T-B-021), and the Xizang Autonomous Region Natural Science Foundation Group Medical Aid Project (No. XZZR202402099(W)).

## Conflict of interests

H.C. and X.Z. are employees of Beijing ClouDNA Co.; the other authors declare no competing financial interests.
