# Supplementary material for: Systematic druggable genome-wide analysis to identify potential therapeutic targets for urologic diseases
Source: Genes Dis. 2025 Nov 8;13(3):101922. doi: 10.1016/j.gendis.2025.101922 (PMC12819038; doi:10.1016/j.gendis.2025.101922)
Supplement: Multimedia component 1 [file mmc1.docx]

**Materials and methods**

## Study design

This study investigates the causal relationship between druggable blood eQTL genes (as exposures) and urologic diseases (as outcomes) using Mendelian randomization. eQTL data were from eQTLGen Consortium and GWAS summary statistics from UK Biobank, with genetic variants associated with the exposure and outcome selected through stringent criteria and harmonized for analysis. As illustrated in Figure S1, a two-sample MR analysis was conducted, followed by heterogeneity and Bayesian colocalization analyses to assess causal effects and shared genetic loci. The therapeutic potential of the identified proteins was explored by evaluating their druggability using the DrugBank database. Functional validation was performed through PPI network and pathway enrichment analysis, while immune-clinical relevance was assessed using the Tumor Cancer Genome Atlas – Bladder Urothelial Carcinoma (TCGA-BLCA) and Gene Expression Omnibus (GEO) datasets, focusing on their role in the immune microenvironment and potential prognostic value for urologic diseases.

## Definition of druggable genes

The set of druggable genes was obtained from Finan et al. (2017)^1^, who redefined the druggable genome by systematically integrating targets of first-in-class drugs approved since 2005, clinical-phase drug candidates, preclinical compounds with protein-binding activity reported in ChEMBL, and genes encoding secreted or membrane proteins amenable to biotherapeutics. In total, 4,479 protein-coding genes were classified as drugged or druggable.

## eQTL data source

Expression quantitative trait loci (eQTL) data were obtained from the eQTLGen Consortium (<https://eqtlgen.org>), a large-scale meta-analysis of cis-eQTL associations^2;3^1;2, comprising 31,684 blood samples. This dataset comprises 31,684 blood samples and includes 16,989 cis-eQTL genes. We specifically selected cis-eQTL, as they represent genetic variants that regulate gene expression levels in close proximity to the target gene, thereby enhancing biological interpretability. Among the 4,479 druggable genes, 2,606 were found to have cis-eQTL evidence (Table S2).

## GWAS summary statistics for urological diseases

Summary-level GWAS data for urological diseases were based on UK Biobank (UKB, <http://www.nealelab.is/uk-biobank/> ), a large-scale prospective cohort study. Urological diseases were defined based on International Classification of Diseases (ICD) codes, including both benign and malignant conditions. Specifically, we included individuals diagnosed with benign urological conditions (e.g., benign prostatic hyperplasia, nephrolithiasis) and urological malignancies (e.g., bladder cancer, prostate cancer). Detailed ICD codes are provided in Table A.1 in Appendix A.

## Select of instrumental variables

For validity of Mendelian randomization (MR) analysis, selected genetic instruments must satisfy the following core assumptions: (1) Relevance Assumption: The genetic variants must be strongly associated with the expression of the target gene. (2) Independence Assumption: The selected variants should be independent of confounders that may influence both gene expression and urological disease risk. (3) Exclusion Restriction Assumption: The effect of the genetic variants on the outcome should be mediated exclusively through gene expression, with no direct pleiotropic effects^43^. The IVW method assumes the validity of all genetic instruments and provides the highest statistical power among all methods when this assumption is met^54^.

To meet these assumptions, genetic variants were carefully selected based on their strong association with the gene, ensuring that they meet the criteria for relevance and independence. Instrumental variables (IVs) were restricted to variants achieving genome-wide significance (P < 1E-6) to ensure robust associations with gene expression. To minimize linkage disequilibrium (LD) and retain independent variants, LD pruning was conducted at r² < 0. 1 within a ±1 Mb window.

## Two-sample mendelian randomization

For causal inference, two-sample Mendelian randomization (MR) analyses were conducted using methodological approaches tailored to the number of available instrumental SNPs. For genes with only two independent SNPs, the inverse-variance weighted (IVW) fixed-effects model was applied, providing efficient estimates under the assumption of minimal pleiotropy^65^. When three or more independent SNPs were available, a comprehensive set of MR methods was employed to mitigate potential biases and heterogeneity. These included MR-Egger regression to assess directional pleiotropy^76^, weighted median estimation to ensure robust effect estimates even when up to 50% of instrumental variables are invalid^87^ and the IVW random-effects model to account for heterogeneity.

All statistical analyses were performed in R using the TwoSampleMR (V0.6.10) packages^98^. Multiple testing correction for MR estimates was applied using the Benjamini-Hochberg method, with statistical significance set at P < 0.05 after correction^10;119;10^. To identify robust and strong correlations, we applied more stringent criteria: (1) adjusted P-values < 0.01; (2) odds ratios (OR) < 0.99 or OR > 1.01. Cochran’s Q methods were used to test for heterogeneity^1211^.

## Colocalization analysis

To further assess whether the identified eQTL signals and GWAS risk loci share a common causal variant, we performed colocalization analysis using the Coloc (V5.2.3) R package^1312^. This Bayesian framework estimates five posterior probabilities (PP₀–PP₄) to evaluate different colocalization scenarios: PP₀, neither gene expression nor the disease is associated with the locus; PP₁, only gene expression is associated with the locus; PP₂, only the disease is associated with the locus; PP₃, both gene expression and the disease are associated, but with different causal variants; PP₄, both gene expression and the disease share a single causal variant (evidence for colocalization).

## Protein-protein interaction network and functional enrichment analysis

The functional role of the identified drug target gene was explored using a protein-protein interaction (PPI) network build with the STRING database (<https://string-db.org/>, V12.0) ^14 13^. The target gene was queried in STRING, and interactions with a combined score > 0.4 (medium confidence) were retained for network construction. The PPI network was visualized and analyzed directly within the STRING platform. Furthermore, Gene Ontology (GO) enrichment analysis was performed using STRING to identify significantly enriched biological processes, associated with the interacting proteins^1514^. Only terms with FDR-adjusted P < 0.05 were considered statistically significant.

## Validation of therapeutic target

To investigate the association between LTK expression and tumor immune evasion in bladder cancer, we employed the Tumor Immune Dysfunction and Exclusion (TIDE) platform (<http://tide.dfci.harvard.edu/>). The analysis was conducted using gene expression data from the Tumor Cancer Genome Atlas – Bladder Urothelial Carcinoma (TCGA-BLCA) cohort, focusing on key immune parameters that reflect T cell activity within the tumor microenvironment. Specifically, the cytotoxic T lymphocyte (CTL) correlation was assessed to determine the relationship between LTK expression and CTL infiltration levels, while the T cell dysfunction score was used to evaluate the extent of immune dysfunction associated with LTK expression.

The association between LTK expression and clinical prognosis in bladder cancer was analyzed with the GSE154261 dataset from the Gene Expression Omnibus (GEO) database, which includes transcriptomic profiles and corresponding clinical data. Patients were stratified into LTK-high and LTK-low groups based on gene expression levels. Kaplan–Meier (KM) survival analysis was performed to estimate recurrence-free survival (RFS) differences between the groups, and statistical significance was assessed using the log-rank test. P-value < 0.05 was considered statistically significant. In addition, the GSE119195 BPH RNA-seq dataset was used to investigate the role of CCNA2 in benign prostatic hyperplasia (BPH).

## Druggability assessment

The DrugBank database (V5.1.13; <https://go.drugbank.com/>) was queried to identify existing therapeutics and compounds in development related to candidate genes. The search criteria included both approved and investigational drugs, focusing on those with evidence of interaction with the gene products at the molecular level. Additionally, [ClinicalTrials.gov](http://clinicaltrials.gov/) (<https://clinicaltrials.gov/>) was searched to identify ongoing or completed clinical trials targeting the identified genes or their associated pathways.

**Reference**

1. Finan C, Gaulton A, Kruger FA, et al. The druggable genome and support for target identification and validation in drug development. *Sci Transl Med.* 2017;9(383).

2. Wang D, Liu S, Warrell J, et al. Comprehensive functional genomic resource and integrative model for the human brain. *Science.* 2018;362(6420).

3. Vosa U, Claringbould A, Westra HJ, et al. Large-scale cis- and trans-eQTL analyses identify thousands of genetic loci and polygenic scores that regulate blood gene expression. *Nat Genet.* 2021;53(9):1300-1310.

4. Davies NM, Holmes MV, Davey Smith G. Reading Mendelian randomisation studies: a guide, glossary, and checklist for clinicians. *BMJ.* 2018;362:k601.

5. Burgess S, Davey Smith G, Davies NM, et al. Guidelines for performing Mendelian randomization investigations: update for summer 2023. *Wellcome Open Res.* 2019;4:186.

6. Perry BI, Burgess S, Jones HJ, et al. The potential shared role of inflammation in insulin resistance and schizophrenia: A bidirectional two-sample mendelian randomization study. *PLoS Med.* 2021;18(3):e1003455.

7. Bowden J, Davey Smith G, Burgess S. Mendelian randomization with invalid instruments: effect estimation and bias detection through Egger regression. *Int J Epidemiol.* 2015;44(2):512-525.

8. Bowden J, Davey Smith G, Haycock PC, Burgess S. Consistent Estimation in Mendelian Randomization with Some Invalid Instruments Using a Weighted Median Estimator. *Genet Epidemiol.* 2016;40(4):304-314.

9. Hemani G, Zheng J, Elsworth B, et al. The MR-Base platform supports systematic causal inference across the human phenome. *Elife.* 2018;7.

10. Liu M, Yu D, Pan Y, et al. Causal Roles of Lifestyle, Psychosocial Characteristics, and Sleep Status in Sarcopenia: A Mendelian Randomization Study. *J Gerontol A Biol Sci Med Sci.* 2024;79(1).

11. Yuan S, Larsson SC. An atlas on risk factors for type 2 diabetes: a wide-angled Mendelian randomisation study. *Diabetologia.* 2020;63(11):2359-2371.

12. Greco MF, Minelli C, Sheehan NA, Thompson JR. Detecting pleiotropy in Mendelian randomisation studies with summary data and a continuous outcome. *Stat Med.* 2015;34(21):2926-2940.

13. Giambartolomei C, Vukcevic D, Schadt EE, et al. Bayesian test for colocalisation between pairs of genetic association studies using summary statistics. *PLoS Genet.* 2014;10(5):e1004383.

14. Szklarczyk D, Kirsch R, Koutrouli M, et al. The STRING database in 2023: protein-protein association networks and functional enrichment analyses for any sequenced genome of interest. *Nucleic Acids Res.* 2023;51(D1):D638-D646.

15. Gene Ontology C. Gene Ontology Consortium: going forward. *Nucleic Acids Res.* 2015;43(Database issue):D1049-1056.

**Supplementary Information**

**Table S1. ICD-10 codes and description of urologic diseases included as outcomes.**

| ICD-10 code | Description |
| --- | --- |
| C61 | Malignant neoplasm of prostate |
| C64 | Malignant neoplasm of kidney, except renal pelvis |
| C67 | Malignant neoplasm of bladder |
| D30 | Benign neoplasm of urinary organs |
| D41 | Neoplasm of uncertain or unknown behaviour of urinary organs |
| N02 | Recurrent and persistent haematuria |
| N10 | Acute tubulo-interstitial nephritis |
| N12 | Tubulo-interstitial nephritis, not specified as acute or chronic |
| N13 | Obstructive and reflux uropathy |
| N17 | Acute renal failure |
| N18 | Chronic renal failure |
| N19 | Unspecified renal failure |
| N20 | Calculus of kidney and ureter |
| N21 | Calculus of lower urinary tract |
| N23 | Unspecified renal colic |
| N30 | Cystitis |
| N31 | Neuromuscular dysfunction of bladder, not elsewhere classified |
| N35 | Urethral stricture |
| N40 | Hyperplasia of prostate |
| N41 | Inflammatory diseases of prostate |
| N43 | Hydrocele and spermatocele |
| N45 | Orchitis and epididymitis |
| N47 | Redundant prepuce, phimosis and paraphimosis |
| N49 | Inflammatory disorders of male genital organs, not elsewhere classified |

ICD, International Classification of Diseases.

Table S2 (Microsoft Excel format). Druggable genes with available cis-eQTLs.


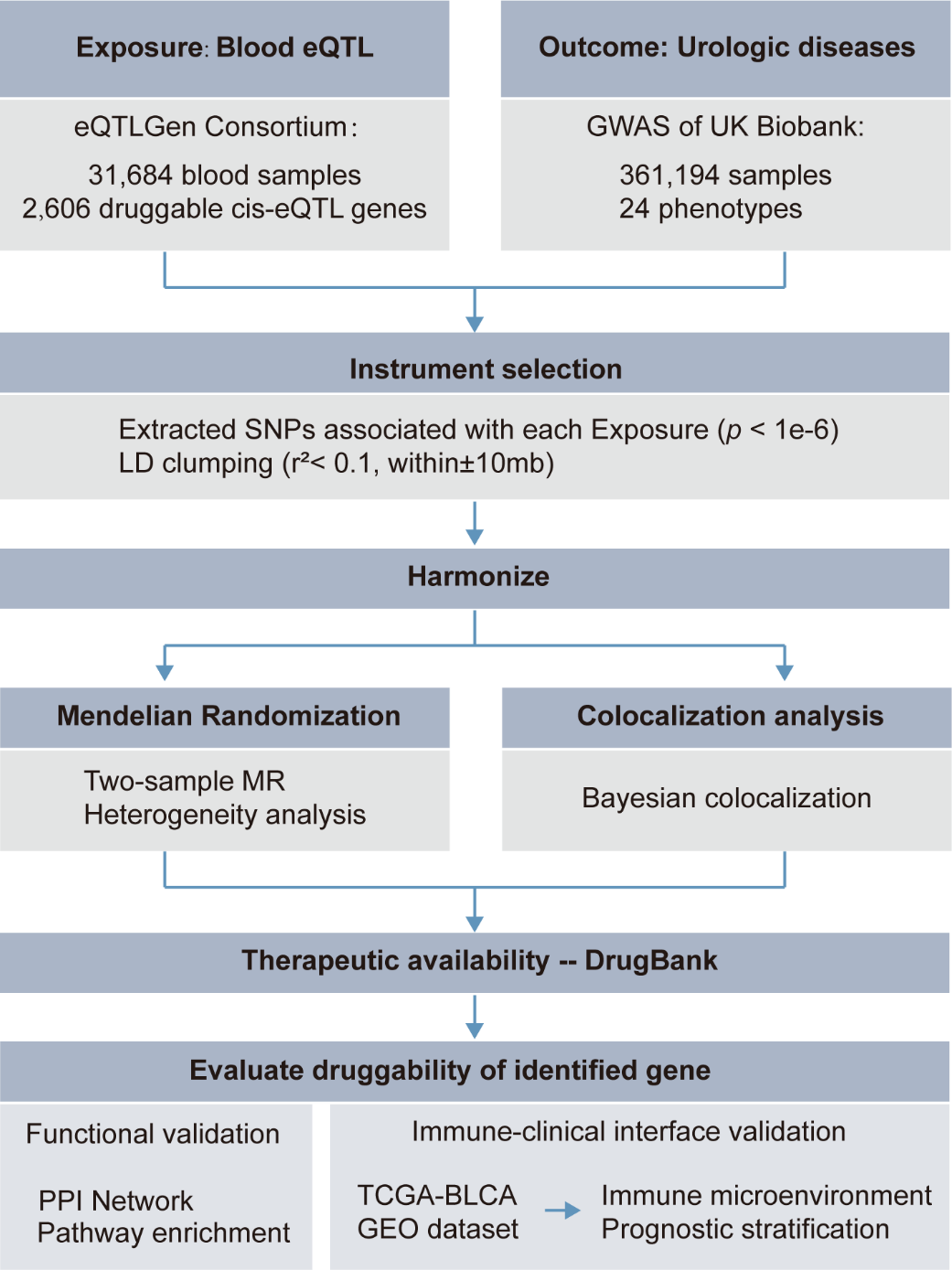


**Figure S1. Flowchart illustrating the two-sample MR analysis between blood eQTLs and urologic diseases.** eQTL, expression quantitative trait loci; cis-eQTL, cis-acting expression quantitative trait loci; GWAS, Genome Wide Association Studies; SNP, single nucleotide polymorphism; LD, linkage disequilibrium; MR, Mendelian randomization; PPI, protein-protein interaction; TCGA, The Cancer Genome Atlas; BLCA, Bladder Urothelial Carcinoma; GEO, Gene Expression Omnibus.**
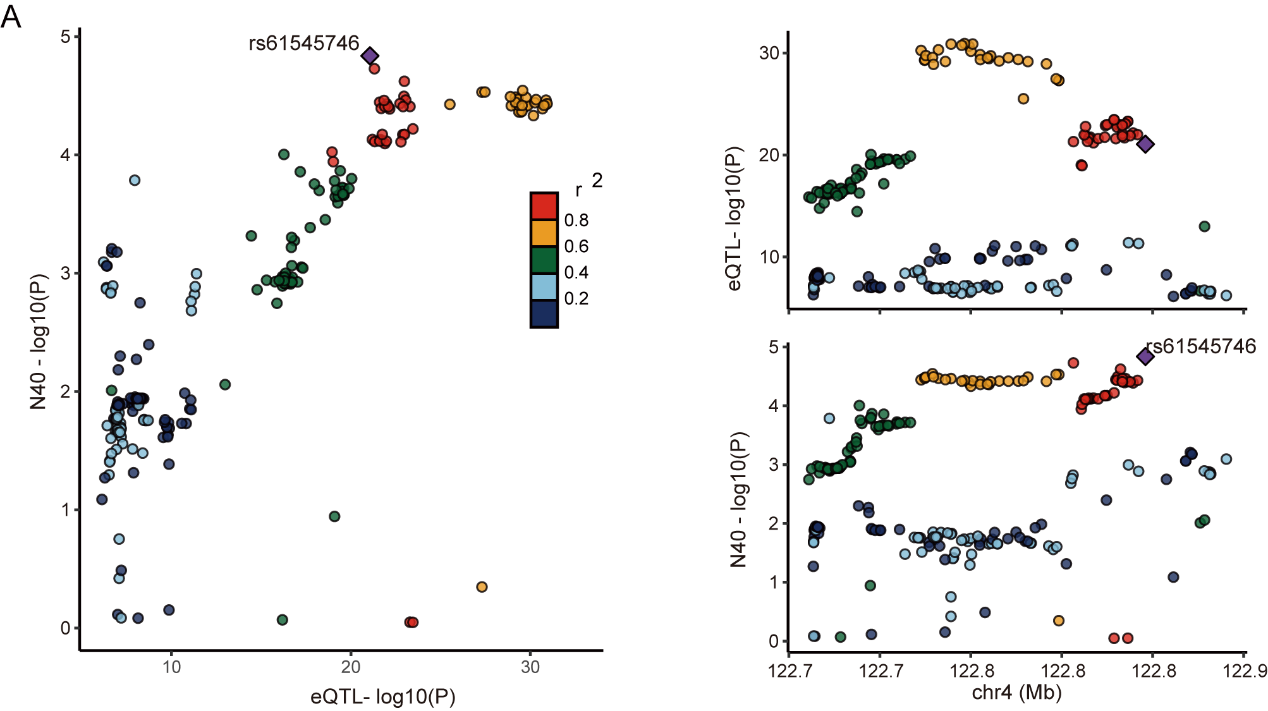
**

**Figure S2. Colocalization analysis of CCNA2 and BPH(N40).**

**
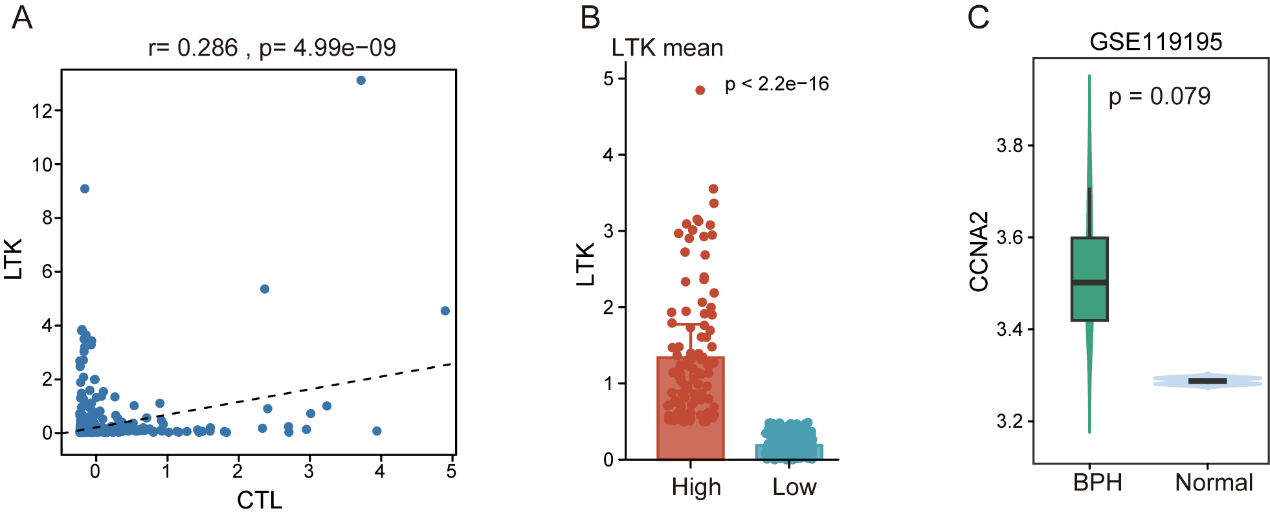
**

**Figure S3. Validation of LTK and CCNA2 expression using bulk RNA-seq data from TCGA and GEO datasets.** (A) Linear association between LTK expression and CTL infiltration in TCGA-BLCA dataset. (B) Differential expression of LTK between groups stratified by its median expression in TCGA-BLCA dataset. (C) Expression levels of CCNA2 in BPH and normal prostate tissues from the GSE119195 dataset.
